# Evaluation of yield, nutritional quality, and Se distribution in black-grained wheat and bioavailable Se concentrations in soil under irrigation and Se fertilizer application

**DOI:** 10.3389/fpls.2025.1521113

**Published:** 2025-03-28

**Authors:** Tianqi Meng, Shuhua Huang, Yinghan Yu, Zhaoxin Sun, Jun Wu, Zahid Akram, Zhengmao Zhang, Yuxiu Liu

**Affiliations:** ^1^ College of Agronomy, Northwest A&F University, Yangling, China; ^2^ Hybrid Rapeseed Research Center of Shaanxi Province, Yangling, China; ^3^ Department of Plant Breeding & Genetics, Pir Mehr Ali Shah Arid Agriculture University, Rawalpindi, Pakistan

**Keywords:** irrigation, yield-related traits, nutritional quality, Se ore powder, black-grained wheat

## Abstract

In the future, ensuring the food and nutritional security of a rapidly growing population will pose an immense challenge in the future. To enhance crop nutrition and address this challenge, a two-year field experiment was conducted on selenium (Se)-deficient dryland soil; the effects of irrigation after Se ore powder (2160 g·ha^−1^) (Se_2160_) application on yield-related traits, nutritional quality, and Se uptake and accumulation in black-grained wheat (BGW) and soil Se availability in soil were investigated. This study aimed to determine whether the combination of Se ore powder application and irrigation enhanced yield-related factors and increased the related nutrient in wheat, thereby achieving biofortification. Irrigation had little effect on the grain protein, amylose, amylopectin, total starch, or soluble sugar content, copper concentration in grains, or the Se translocation factor from the root to grain following Se_2160_ application, but significantly increased the sucrose content and iron (Fe) concentration in grains. Se was readily taken up by roots of irrigated plants in Se_2160_-treated soils, resulting in leaf and grain Se concentrations that were 4–7 times higher than in control soils. Se fertigation increased the Se distribution in the leaves and grains of BGW due to its decline in the roots and spike-stalk + glume. Se_2160_ application significantly increased the grain yield and Fe, zinc, Se and manganese concentrations in grains under water regimes. Bioavailable Se concentrations in the 0–20-cm layer of Se_2160_-treated soil were significantly decreased by irrigation and increasing irrigation amount, but significantly higher than those of control soils, while those in the 20–40-cm layer were not affected. These findings indicate that Se fertigation enhances grain yield, sucrose content, Fe concentration, and Se accumulation in BGW as well as bioavailable Se concentrations in the 0–20-cm soil layer, effects that are conducive to Se-enriched agricultural production and human health improvement.

## 1 Introduction

Food security is the cornerstone of a country’s stable development. In addition to food availability, nutrition is an integral component of food security (Han et al., 2023). Wheat is one of the world’s most important staple crops, providing at least 20% of food calories globally, but the mineral element concentration in its grains is relatively low, and more attention has been given to the breeding of high-yielding cultivars (Maltzahn et al., 2021). An estimated 50% of the global population does not suffer from hunger but rather from the debilitating effects of an unhealthy diet, resulting in malnutrition effects, such as undernutrition, obesity, and micronutrient deficiencies (Padhy et al., 2022). Moreover, with regard to health awareness, people generally prefer nutritionally balanced diets over conventional high-energy diets. Considering food security, major wheat breeding programs have shifted toward a combination of quantity and quality, and color-grained wheat have become a novel option for targeting malnutrition.

Compared to common wheat, which has white or red grains, color-grained wheat is characterized by having grains of various colors, including green, blue, purple, and black. This has attracted attention in nutrition as a functional food due to significant anthocyanin and essential nutrient concentrations, such as total phenolic acid content and iron (Fe) and zinc (Zn) concentrations (Padhy et al., 2022). Black-grained wheat (BGW) is a rich source of protein and micronutrients and a good raw material for value-added products (Liu et al., 2021c). Due to its additional health benefits, BGW has piqued the interest of breeders, consumers, and policymakers as a means to address malnutrition in vulnerable populations. However, BGW is generally inferior to white-grained wheat in terms of yield and its components (Liu et al., 2021b). Thus, the enhancement of grain yield and nutrition in BGW could open a new avenue for providing additional value for this wheat and its derived products, thereby contributing to global food security for an ever-growing population.

Selenium (Se) is an essential micronutrient for humans due to its role in physiological functions (Gupta et al., 2021). Plant-derived dietary Se is the primary source of Se in the body. According to the USDA, in many areas of the world, the Se intake from food consumption is below the recommended dose (55–220 μg·day^−1^), including in the developing countries of Asia and Africa (7–11 μg·day^−1^) (Gupta et al., 2021) and regions in China (less than 40 μg·day^−1^) (Luo et al., 2021). An estimated one billion people globally (Jones et al., 2017) and more than 70 million people in China suffer from Se deficiency (Li et al., 2014). Moreover, the development of diseases, such as Keshan disease and cardiovascular disease, are associated with serious Se deficiency (Yang et al., 2023). Therefore, adequate Se nutrition is important for human health. The micronutrient profiles of edible crops can be improved through agronomic biofortification (Bindu et al., 2024), which can increase the amount of available Se in the edible parts of food crops through fertilizer application to soil, by foliar spraying, or as a seed treatment, thus improving the contents of specific micronutrients (Sarwar et al., 2020; Liu et al., 2021b).

The goal of agronomic biofortification is to achieve an optimum Se concentration in the edible parts of crop plants, but this process is affected by a series of factors. Generally, the Se concentration in the edible portion of the plant is closely correlated with the Se concentration in the soil (Fordyce et al., 2000). The terrestrial distribution of Se is uneven, and the Se concentration ranges from 0.01 to 2.00 mg·kg^−1^in most soils, with an average of 0.40 mg·kg^−1^ (Fordyce, 2013). The soil Se concentration in mainland China varies from 0.01 to 16.24 mg·kg^−1^, with a median of 0.171 mg·kg^−1^ (Liu et al., 2021a), but areas with Se less than 0.40 mg·kg^−1^ account for 72% of the total area in China (Li et al., 2014). Biofortification has been successfully applied in several countries in areas with low Se soils (White, 2016; Sarwar et al., 2020). Selenite (Se^4+^) and selenate (Se^6+^) are the major Se forms for plant absorption and utilization due to their high solubility in soil (Gupta et al., 2021). Previous studies have shown that Se in Se ore powder is mainly present as Se^4+^ (Deng et al., 2018). The application of Se ore powder to the soil significantly increases the Se concentration in different parts of rice, soybean, and wheat plants (Deng et al., 2018; Liu et al., 2021b). Moreover, the concentrations of heavy metals, such as lead (Pb), arsenic (As), mercury (Hg), and chromium (Cr), in brown rice, soybean seeds, and wheat grains were below the limits of detection, and the organic Se accounted for from 72.4%–96% of the total Se in crops after Se ore powder application (Deng et al., 2018; Liu et al., 2021b). Furthermore, the application of Se ore powder to the soil improved wheat growth and Zn, Fe, and manganese (Mn) concentrations in grains (Liu et al., 2021b). Therefore, the use of Se ore powder may be an effective approach for Se biofortification of wheat, compromising between its effectiveness for absorption and its performance in organic Se enrichment grain with Se forms that are more beneficial for humans. However, when applied to the soil, the effectiveness of Se fertilizers is largely dependent on soil properties, such as pH, organic acids, and organic matter content, factors that affect Se absorption and uptake by plants (Guo et al., 2022).

Flood irrigation accelerates Se loss in soil and decreases soil Se bioavailability (Song et al., 2020). Studies have shown that irrigation with Se-enriched water increases the Se content in green bean, cabbage, potato and tomato (Ragályi et al., 2021). However, similar Se concentrations in leaf tissues of yellow sweet clover have been observed under full and limited irrigation (Kostopoulou et al., 2010). Continual flooding increased the amount of total Se in kernels by an average of 90% compared to values measured in rice irrigated using a sprinkler system (Spanu et al., 2020). Irrigation at 80% combined with Se-enriched organic fertilizer increased the Se and vitamin C contents in tomato (Huang et al., 2021). The Se content in the edible parts of plants, such as green bean and carrot, increased up to 75-fold using irrigated water with a concentration of 100 μg Se·L^−1^ (Ragalyi et al., 2022). Different irrigation techniques and crop genotypes are valuable tools for modulating the Se concentration in crop grains in accordance with the needs of different populations. In addition, Se deficiency in dryland soil coupled with water supply deficiency restricts wheat growth and grain yield and affects Se uptake and accumulation by plants. Therefore, Se fertigation may promote BGW growth and Se absorption and utilization. This needs to be confirmed in further investigations because it is unclear how the combined application of Se ore powder and irrigation affects the grain yield and grain nutritional quality of wheat.

The objectives of this study were to investigate the effects of applying Se ore powder to soil (0 and 2160 g·ha^−1^) under three water regimes (no irrigation (W_0_), irrigation at the wintering stage (W_1_), and irrigation at the wintering and green-turning stages (W_2_)) on the net photosynthetic rate (Pn), grain yield and its components, nutritional components, microelement concentrations (Fe, Zn, Mn and Cu), and Se uptake and accumulation in BGW in a 2-year field experiment. The results of this study could provide an effective approach for achieving high-yield and high-nutritional quality wheat through biofortification measures. We hypothesized that Se fertigation would affect the yield and nutrition performance of BGW because Se mobility in the soil varies with water conditions.

## 2 Materials and methods

### 2.1 Plant materials

Two BGW breeding lines were used for this study. Xihei 88 (BGW-1) and Heidali (BGW-2). These materials were bred and/or preserved in our laboratory (College of Agronomy, Northwest A&F University, Shaanxi, China).

### 2.2 Field experiment

Field experiments were conducted for 2 seasons (2018–2019 and 2019–2020) at the research farm of Northwest A&F University, Yangling, Shaanxi, China (34°17’38”N, 108°4’23” W; elevation 525 m). The soil is classified as Loess loam.

The experiment was set up in a split–split plot design with three replicates. The main plot factors were the control with no Se application (Se_0_) and application of 2160 g·ha^−1^Se ore powder (Se_2160_) to the soil. Subplot factors were three water regimes: no irrigation throughout the entire growing period (W_0_); irrigation at the wintering stage (Feekes 3.0) (W_1_); and irrigation at both at the wintering (Feekes 3.0) and green-turning stages (Feekes 4.0) (W_2_). Our previous work with 51.3 mg·kg^−1^ Se ore powder with applied Se concentrations (pure Se) of 1080–4320 g·ha^−1^ showed that 2160 g·ha^−1^ Se application had the greatest effect on grain yield and Fe concentration and increased the organic Se concentration in grains to 0.31–0.35 mg·kg^−1^ (Liu et al., 2021b). Therefore, 2160 g·ha^−1^ Se ore powder was applied before plowing, and the Se concentration in the soil (0–20 cm) was measured to be 0.65 ± 0.05 mg·kg^−1^. Before sowing, 600 kg·ha^−1^ of compound fertilizer (20-20-6, Summit Fertilizer [Qingdao] Co., Ltd., China) containing 20% N, 20% P_2_O_5_, and 5% K_2_O was applied to each plot (9.0×1.2 m^2^).

**Figure 1:**
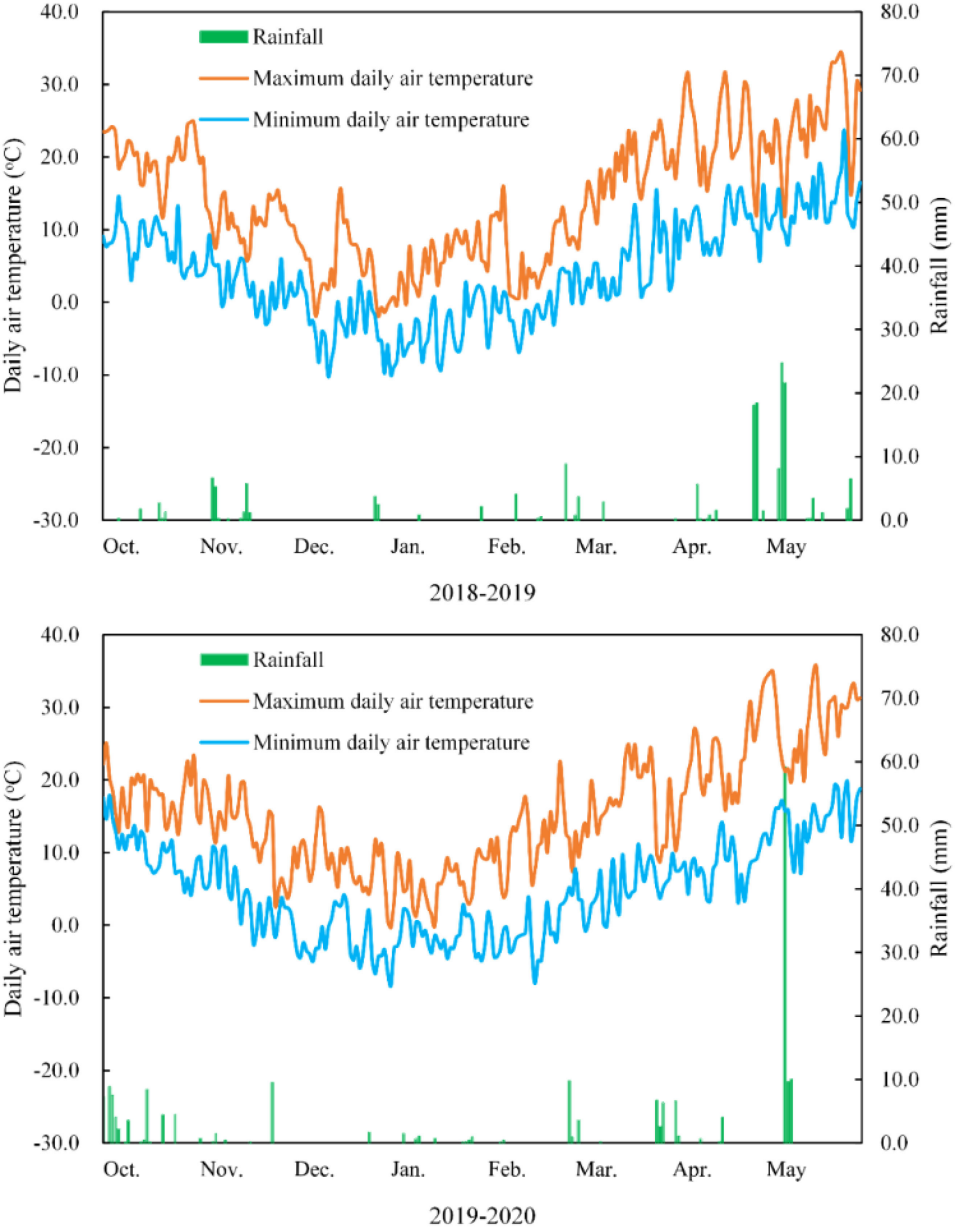
Maximum and minimum daily air temperatures at 2 m and rainfall amount in 2 wheat growing seasons.

The rainfall and daily air temperature were recorded throughout the growth period in the two seasons and are shown in **Figure 1**. The rainfall was 169.4 mm and 192.5 mm for the 2 growing seasons. Irrigation was provided using tap water at the wintering stage (60 mm, December 30, 2018 and 2019) and in the greening stages (60 mm, March 15, 2018 and 2019). Herbicides, fungicides, and insecticides were applied whenever necessary. Seeds were sown on October 5, 2018 and October 7, 2019, and plants were harvested on June 2, 2019, and June 5, 2020.

### 2.3 Soil element content

According to the Soil Physicochemical Analysis Handbook (Bao, 2000), before the soil was turned, 20 cm of soil was obtained from a soil sample. After sifting with a 1-mm sieve, 0.2 g was collected, and 6 mL of concentrated sulfuric acid was added. The mixture was then digested with a microwave digester (Mutiwave PRO, Anton Paar, Austria). The deboiling tube was placed on the automatic Kay-type nitrogen analyzer for distillation and titration, and the total nitrogen content of the soil was determined. The boiled liquid was diluted to 50 mL and filtered with a 0.2-um filter membrane, and the Se content was determined using an inductively coupled plasma mass spectrometer (iCAP RQ, Thermo Fisher, USA).

A 0.5-g of soil sample was passed through a 0.15-mm sieve, and 5 mL of 0.800 mol·L^−1^ of 1/6 K^2^Cr^2^O^7^ solution and 5 mL of concentrated H^2^SO^4^ solution were added and boiled at 180°C for 5 min. The liquid was transferred, and the volume was adjusted to 70 mL, two drops of 1,10-phenanthroline were added, and the solution was titrated with 0.2 mol·L^−1^ of FeSO^4^ solution until a brick red color appeared. The titration volume was recorded, and a control experiment was performed with powdered SiO^2^ instead of soil. The following formula was used to calculate the soil organic matter content(Os, g·kg^-1^):


$$Os=\frac{V_{0}-V}{24\left(1-H\right)}\times 1.1\times 1.724$$

where $V_{0}$ is the volume of FeSO_4_ used in the titration (mL); V is the volume of FeSO_4_ used in the titration of the soil sample (mL); 1.1 is the oxidation correction factor; and 1.724 is the conversion factor

Then, 2.5-g soil samples were screened using a screen with an aperture of 1 mm, and 0.50 mol·L^−1^ NaHCO^3^ and 1 g of phosphorus-free activated carbon were added. The samples were oscillated at 180 r·min^−1^ for 30 min at 25°C and filtered with phosphorus-free filter paper. A 5-mL volume of a molybdenum-antimony inhibitor was added to 10 mL of filtrate and incubated in a water bath at 40°C for 30 min. The absorbance was measured at wavelength 700 nm using colorimetry, and a standard curve was generated. The soil available phosphorus content (Pa, mg·kg^-1^) was calculated as follows:


$$Pa=\frac{0.50\times \rho }{1-H}$$

where ρ is the mass concentration of phosphorus on the standard curve (μg·mL^−1^) and H is the moisture content of the soil sample (%).

Soil samples weighing 5 g were screened using a screen with a 1-mm aperture, and 50 mL of 1mol·L^−1^ ammonium acetate solution was added. The samples were oscillated at 180 r·min^−1^ for 30 min at 25°C and filtered with filter paper, and the content of available potassium content was determined with a flame photometer (M410, Sherwood, UK).

Soil samples weighing 10 g were screened using a screen with a 1-mm aperture, and 25 mL of distilled water was added to make a soil suspension. The pH value of the soil solution was measured with a pH meter.

The soil contained 9.7 g·kg^−1^organic matter content, 1.5 g·kg^−1^ total N, 10.5 mg·kg^−1^ available P, 250.1 mg·kg^−1^ available K, and 0.24 mg·kg^−1^ total Se and had a pH of 8.2.

### 2.4 Determination of yield-related traits

Five flag leaves of wheat were labeled and sampled. Net photosynthetic rate (Pn) was measured in the field between 9:00 am and 11:00 am during stages of anthesis (Feekes 10.5.3–5.4), early grain-filling (GF-1, Feekes 11.1), and mid grain-filling (GF-2, Feekes 11.1) (Miller, 1999) using a portable photosynthesis system (LI-6400, LI-COR Inc. Lincoln, NE, USA). Measurements were taken in a 6-cm^2^ leaf chamber under ambient CO_2_ concentration of 380–400 μmol CO_2_·mol^-1^, a radiation of 1400 μmol·m^−2^ s^−1^, and a leaf temperature of 28°C.

Plants were sampled in a 1-m^2^ plot with four rows for yield components. Spike numbers were measured from the samples and then transformed to spike number per m^2^. Kernel number (spike^−1^) was calculated as the average of ten spikes randomly selected from the samples. Plots were harvested at the maturity stage (Feekes 11.3–11.4) by small-plot combines (4LXNK-1.0, Weihui Xinnongke Machinery Factory, China). Grain yield (t·ha^−1^) was determined from the harvested weight adjusted to a 12% moisture content. Thousand kernel weight (g) was measured using 1,000 grains from the harvested samples in each plot.

### 2.5 Determination of grain nutritional components

The N content of grains was measured according to the Kjeldahl nitrogen determination method (AACC, 2000) with an automatic nitrogen determination analyzer (Kjeltec 8400, FOSS). Grain protein content (%) was calculated by multiplying the N content by a coefficient of 5.7.

Amylose and amylopectin fractions from wheat starch were isolated and obtained using the butanol precipitation method as reported previously (Zhu et al., 2008). The amylose and amylopectin contents were determined by dual-wavelength iodine binding colorimetry (Zhu et al., 2008). Total starch content was the sum value of amylose and amylopectin content.

A 0.2-g sample of dried grain powder was extracted using 6.0 ml of 80% (v/v) ethanol in a water bath at 80°C for 30 min and centrifuged at 5000*×g* for 10 min. This extraction procedure was performed three times, and the supernatants were collected, mixed, and then diluted with 80% ethanol to 25.0 mL for the measurement of soluble sugar and sucrose contents according to the protocol as described by Buysse and Merckx (1993).

### 2.6 Determination of Fe, Zn, Mn, Cu, and Se concentrations in grains

After harvest, the plants were washed with tap water and then with deionized water to remove dust and soil. Subsequently, the washed plants were separated into the various parts (grain, spike-stalk + glume, leaves, stem + leaf sheath, and root) and then dried in a forced–air oven at 75°C to a constant weight. The oven–dried samples were ground into fine powder using a freezing mixer mill (MM400, Retsch, Haan, Germany) after passing through a 100-mesh sieve and then stored in a sealed plastic bag.

A 0.5-g powder sample was placed into the polytetrafluoroethylene digestion tube with 10.0 mL HNO_3_ and 2.0 mL H_2_O_2_ and then was digested in a closed microwave digestion system (MARS6, CEM, Matthews, NC, USA). After cooling to room temperature, the digested solution was transferred to a volumetric flask and diluted with ultrapure water to 25 mL for the determination of Fe, Zn, Mn, and Cu concentrations using atomic absorption spectrometry (PinAAcle 900F, PerkinElmer Enterprise Management Co. Ltd. Waltham, MA, USA) according to the Standard Method GB/T 5009.14-2017 developed by the Ministry of Health of China. About 5.0 mL 6.0 mol·L^−1^ HCl was added to the obtained digested solution, and then the mixed solution was diluted with ultrapure water to 25 mL. The 10.0 mL mixed solution was transferred to a reaction vessel, with 2.5 mL 100 g·L^−1^ K_3_Fe (CN)_3_ being added for total Se concentration in grains determination using a liquid-phase atomic fluorescence spectrometer (LC-AFS9780, Beijing Haiguang Instrument Co. Ltd. Beijing, China) according to the Standard Method GB/T 5009.93-2017 developed by the Ministry of Health of China.

### 2.7 Determination of the Se distribution and accumulation

Se distribution and accumulation in each part were calculated as follows:


$$D_{Se}=\frac{C_{i}\;\times \;D_{w}}{A_{w}}$$

where $D_{Se}$ is the proportion of Se distributed in the grain, spike-stalk + glume, leaves, stem + leaf sheath and root (%); $C_{i}$ is the Se concentration in the above-mentioned part (mg·kg^−1^); $D_{w}$ is the dry weight of above-mentioned parts (kg·plant^−1^); and $A_{w}$ is the Se concentration in the whole wheat plant (mg·plant^−1^).

The translocation factor (TF) of Se from the root to grain ( $TF_{root\;-grain}$ ) was calculated as follows:


$$TF_{root\;-grain}\;=\;\frac{C_{grain}}{C_{root}},$$

where $C_{grain}$ is the Se concentration in the above-mentioned part (mg·kg^−1^); $D_{root}$ is the Se concentration in the above-mentioned part (mg·kg^−1^).

### 2.8 Determination of soil bioavailable Se concentrations

Soil samples were collected at depths of 0–20 cm and 20–40 cm from the experimental site and then air-dried at room temperature. After removing plant residues, the soil samples were homogenized and sieved through a 100-mesh for measuring the soil bioavailable Se concentration according to the method described by Li et al. (2016).

A 1.0-g amount of soil was added into a 100-mL centrifuge tube and extracted with 10.0 mL 0.25 mol·L^−1^ KCl (soil/liquid = 1:10) by shaking at 200*×g* for 1 h at 25°C. Afterward, the mixture was centrifuged at 3000*×g* for 10 min and filtered through a 0.45-μm filter. The supernatant was collected for soluble Se determination. The residue in the above tube was continuously extracted with 10.0 mL 0.7 mol·L^−1^ KH_2_PO_4_ (pH 5.0) and shaken at 200*×g* and 25°C for 4 h. Then, the mixed solution was centrifuged at 3000×*g* for 10 min and filtered. The collected supernatant was used for exchangeable Se determination. The soil available Se concentration value was the sum of soluble Se and exchangeable Se concentration.

### 2.9 Data analysis

The data were recorded as mean values ± standard deviations (SD). The statistical analysis was carried out by analysis of variance (ANOVA) procedures using JMP V12.0 statistical software from SAS (version 9, SAS Institute, Inc. Cary, NC, USA). Significant differences among water regimes as well as between Se_0_ (without Se added) and Se_2160_ (2160 g·ha^−1^ Se ore powder added) were detected using Fisher’s protected LSD at α = 0.01 and α = 0.05, respectively.

## 3 Results

### 3.1 Effects of irrigation and Se application on yield-related traits

**Figure 2:**
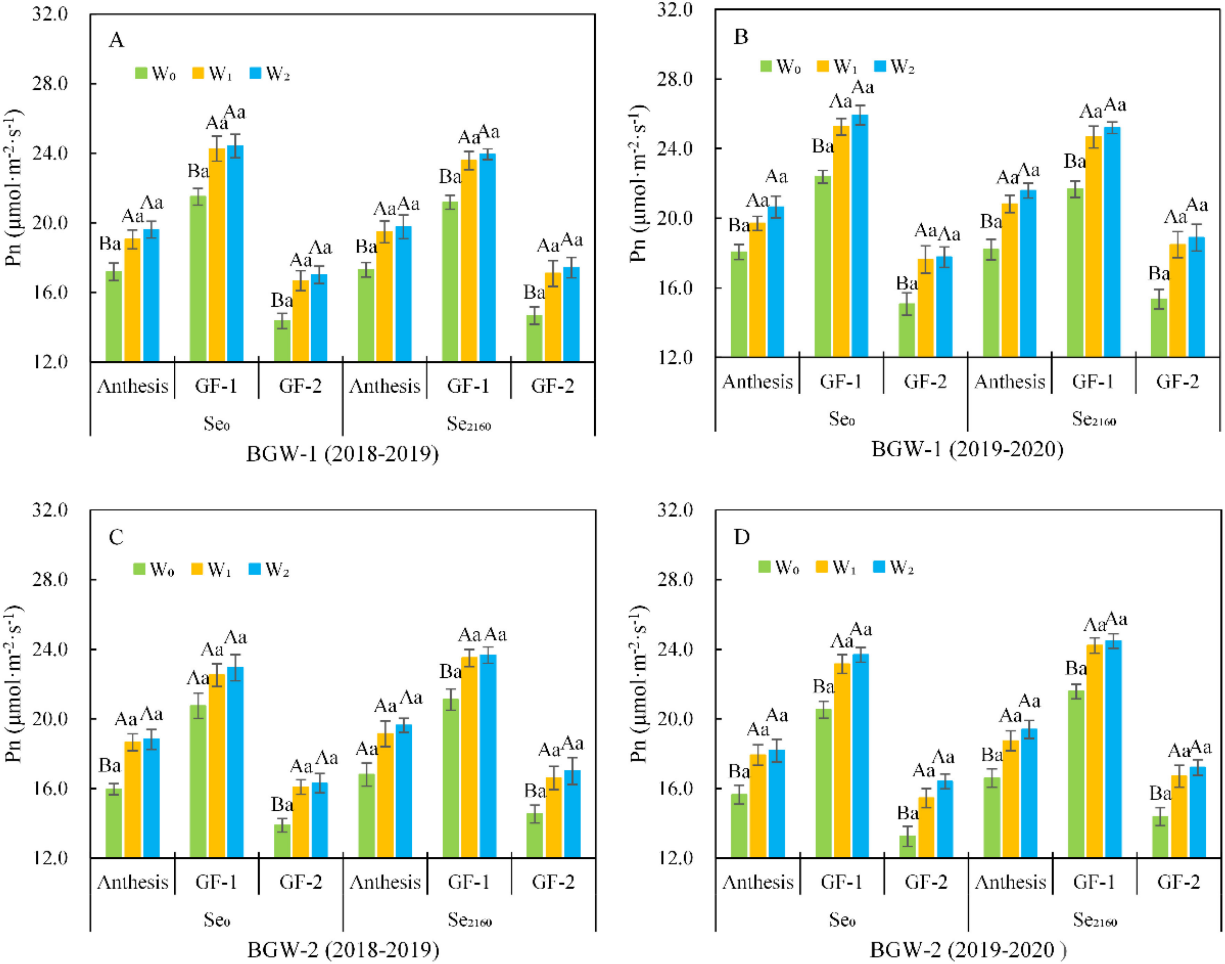
Effect of irrigation on the Pn in black-grained wheat **(AB** and **CD)** at the post-anthesis in soil with different Se contents in two seasons **(AC** and **BD)**. For a given Se treatment, bars labeled with different capital letters indicate significant differences at α = 0.01 among three water treatments at a given stage; for a given stage, bars labeled with the different lowercase letter(s) indicate significant differences at α = 0.05 between two Se treatments at a given water treatments.W_0_, no irrigation throughout the entire growing period; W_1_, irrigated at the wintering stage (Feekes 3.0); W_2_, irrigated at both the wintering and greening stage (Feekes 4.0); Se_0_, no Se fertilizer; Se_2160_, 2160 g·ha^-1^ pure Se; BGW-1, black-grained wheat Xihei 88; BGW-2, black-grained wheat Heidali; anthesis (Feekes 10.5.2), anthesis stage, GF-1, early grain-filling stage (Feekes 10.5.4); GF-2, mid grain-filling stage (Feekes 11.1).

As shown in **Figure 2**, the net photosynthetic rate (Pn) of BGW (BGW-1 and BGW-2) initially significantly increased initially and then decreased from anthesis to the GF-2 stage, and its highest value was observed at the GF-1 stage in both seasons. After soil application of Se_0_ and Se_2160_, irrigation significantly increased the Pn of BGW at the anthesis, GF-1, and GF-2 stages (α = 0.01). There were no significant differences in Pn between W_1_ and W_2_ treatments. Within the same water regime (W_0_, W_1_, or W_2_), the Se_2160_ fortification did not result in any significant change in Pn at each stage compared to control samples with no Se fortification.

Grain yield and its components increased with increasing irrigation amounts in both seasons (**Table 1**). Regardless of the Se treatment, BGW had a significantly higher grain yield, spike number, and kernel number under W_2_ treatment than under W_0_ treatment (α = 0.01), but no significant differences were observed between W_1_ and W_0_ treatments. In addition, there were no significant differences in the 1000-kernel weight among the 3 water regimes. Se_2160_ application significantly increased the grain yield, kernel number, and thousand kernel weight, compared to Se_0_ application (α = 0.05), but no significant differences were observed for spike number under the three water regimes (W_0_, W_1_, and W_2_).

**Table 1:** Effect of irrigation on the grain yield and yield components of black-grained wheat in different Se soil in two seasons.

Seasons	Treatments	Grain yield (t·ha^-1^)				Spike number (m^2^)			
		BGW-1		BGW-2		BGW-1		BGW-2	
		Se_0_	Se_2160_	Se_0_	Se_2160_	Se_0_	Se_2160_	Se_0_	Se_2160_
2018-2019	W_0_	4.39 ± 0.15^Bb^	5.09 ± 0.14^Ba^	3.77 ± 0.12^Bb^	4.32 ± 0.14^Ba^	264.6 ± 10.8^Ba^	281.6 ± 10.4^Ba^	339.3 ± 11.5^Ba^	360.0 ± 9.85^Ba^
	W_1_	4.70 ± 0.13^ABb^	5.61 ± 0.15^Ba^	4.04 ± 0.11^Bb^	4.80 ± 0.16^Ba^	288.0 ± 10.1^ABa^	305.7 ± 11.9^ABa^	360.0 ± 10.8^ABa^	383.8 ± 11.8^ABa^
	W_2_	5.29 ± 0.22^Ab^	6.52 ± 0.12^Aa^	4.56 ± 0.13^Ab^	5.56 ± 0.10^Aa^	312.0 ± 13.9^Aa^	332.0 ± 14.4^Aa^	385.1 ± 13.0^Aa^	410.3 ± 10.2^Aa^
2019-2020	W_0_	5.00 ± 0.16^Bb^	5.78 ± 0.15^Ba^	3.98 ± 0.16^Bb^	4.60 ± 0.12^Ba^	275.3 ± 13.0^Ba^	294.7 ± 10.8^Ba^	350.7 ± 9.00^Ba^	372.0 ± 13.9^Ba^
	W_1_	5.32 ± 0.15^ABb^	6.32 ± 0.14^Ba^	4.13 ± 0.15^Bb^	5.11 ± 0.15^Ba^	305.2 ± 7.57^Ba^	324.2 ± 8.40^Ba^	378.2 ± 10.0^Ba^	400.5 ± 11.3^ABa^
	W_2_	5.93 ± 0.27^Ab^	7.28 ± 0.20^Aa^	4.79 ± 0.19^Ab^	5.99 ± 0.19^Aa^	344.0 ± 8.00^Aa^	365.3 ± 4.62^Aa^	416.0 ± 8.00^Aa^	437.3 ± 16.0^Aa^
		Kernel number (spike^-1^)				Thousand kernels weight (g)			
2018-2019	W_0_	59.5 ± 0.85^Bb^	61.5 ± 0.82^Ba^	39.0 ± 0.57^Bb^	41.2 ± 0.51^Ba^	37.5 ± 0.72^Ab^	39.6 ± 0.59^Aa^	37.3 ± 0.81^Ab^	40.1 ± 0.56^Aa^
	W_1_	60.1 ± 0.99^Bb^	63.2 ± 0.56^Ba^	40.2 ± 0.78^Bb^	43.1 ± 0.71^Ba^	38.1 ± 0.64^Ab^	40.9 ± 0.91^Aa^	37.9 ± 0.62^Ab^	41.3 ± 0.59^Aa^
	W_2_	63.9 ± 1.25^Ab^	67.7 ± 0.93^Aa^	43.4 ± 0.76^Ab^	47.4 ± 1.50^Aa^	39.2 ± 0.51^Ab^	42.7 ± 1.30^Aa^	38.9 ± 0.98^Ab^	42.0 ± 0.85^Aa^
2019-2020	W_0_	60.0 ± 0.46^Bb^	63.0 ± 0.77^Ba^	40.1 ± 0.42^Bb^	42.1 ± 0.42^Ba^	37.7 ± 0.75^Aa^	40.2 ± 0.65^Aa^	36.4 ± 0.49^Ab^	39.4 ± 0.68^Aa^
	W_1_	60.3 ± 0.81^Bb^	64.8 ± 1.05^Ba^	40.8 ± 0.64^Bb^	43.7 ± 0.74^Ba^	38.4 ± 0.85^Ab^	41.5 ± 0.80^Aa^	37.1 ± 0.59^Ab^	40.7 ± 0.65^Aa^
	W_2_	64.0 ± 1.59^Ab^	68.2 ± 1.00^Aa^	45.1 ± 1.51^Ab^	48.3 ± 1.22^Aa^	39.6 ± 0.95^Ab^	42.3 ± 0.94^Aa^	38.2 ± 0.68^Ab^	41.5 ± 0.85^Aa^

Values with different capital letter in the same column are significantly different (α = 0.01). Values with different small letters in the same row are significantly different (α = 0.05). W_0_, no irrigation throughout the entire growing period; W_1_, irrigated at the wintering stage (Feekes 3.0); W_2_, irrigated at both the wintering and greening stage (Feekes 4.0); Se_0_, no Se fertilizer; Se_2160_, 2160 g·ha^-1^ pure Se; BGW-1, black-grained wheat Xihei 88; BGW-2, black-grained wheat Heidali.

### 3.2 Effects of irrigation and Se application on nutritional components

After applying Se_2160_ to the soil, irrigation slightly decreased the grain protein and amylose contents and increased the amylopectin, total starch, and soluble sugar contents, but no significant differences were found among the three water regimes in either season (**Table 2**). Following Se_0_ application, the sucrose content in grains was significantly higher under W_2_ treatment than under W_0_ treatment (α = 0.01), but no significant differences were observed between W_1_ and W_0_ treatments. After applying Se_2160_, the sucrose content significantly increased with irrigation and with an increasing irrigation amount (α = 0.01).

**Table 2:** Effect of irrigation on the nutritional components of black-grained wheat in different Se soil in two seasons.

Seasons	Treatments	Grain protein content (%)				Amylose content (%)			
		BGW-1		BGW-2		BGW-1		BGW-2	
		Se_0_	Se_2160_	Se_0_	Se_2160_	Se_0_	Se_2160_	Se_0_	Se_2160_
2018-2019	W_0_	17.21 ± 0.22^Ab^	18.22 ± 0.20^Aa^	14.36 ± 0.18^Ab^	15.48 ± 0.23^Aa^	11.34 ± 0.15^Ab^	13.97 ± 0.19^Aa^	11.79 ± 0.22^Ab^	14.13 ± 0.19^Aa^
	W_1_	17.05 ± 0.15^Ab^	18.07 ± 0.17^Aa^	14.19 ± 0.20^Ab^	15.33 ± 0.20^Aa^	11.31 ± 0.21^Ab^	13.83 ± 0.22^Aa^	11.60 ± 0.15^Ab^	14.12 ± 0.20^Aa^
	W_2_	16.87 ± 0.17^Ab^	17.95 ± 0.19^Aa^	13.97 ± 0.16^Ab^	15.07 ± 0.18^Aa^	11.26 ± 0.18^Ab^	13.81 ± 0.18^Aa^	11.57 ± 0.19^Ab^	14.07 ± 0.23^Aa^
2019-2020	W_0_	16.51 ± 0.15^Ab^	17.53 ± 0.21^Aa^	13.68 ± 0.19^Ab^	14.78 ± 0.19^Aa^	11.92 ± 1.17^Ab^	14.51 ± 0.18^Aa^	12.35 ± 0.16^Ab^	14.74 ± 0.18^Aa^
	W_1_	16.30 ± 0.20^Ab^	17.36 ± 0.16^Aa^	13.48 ± 0.16^Ab^	14.60 ± 0.22^Aa^	11.85 ± 1.18^Ab^	14.39 ± 0.21^Aa^	12.31 ± 0.23^Ab^	14.71 ± 0.17^Aa^
	W_2_	16.09 ± 0.19^Ab^	17.13 ± 0.18^Aa^	13.26 ± 0.21^Ab^	14.30 ± 0.18^Aa^	11.77 ± 1.18^Ab^	14.36 ± 0.20^Aa^	12.29 ± 0.17^Ab^	14.66 ± 0.19^Aa^
		Amylopectin content (%)				Total starch content (%)			
2018-2019	W_0_	51.78 ± 0.92^Ab^	54.36 ± 1.02^Aa^	54.12 ± 1.04^Ab^	56.79 ± 1.02^Aa^	63.12 ± 0.87^Ab^	68.33 ± 0.91^Aa^	65.81 ± 0.95^Ab^	70.92 ± 1.01^Aa^
	W_1_	52.60 ± 1.04^Ab^	55.74 ± 1.00^Aa^	55.04 ± 1.03^Ab^	58.12 ± 1.05^Aa^	63.91 ± 1.04^Ab^	69.57 ± 1.03^Aa^	66.64 ± 1.08^Ab^	72.24 ± 1.03^Aa^
	W_2_	53.91 ± 1.02^Ab^	57.27 ± 1.01^Aa^	56.37 ± 0.90^Ab^	59.61 ± 1.03^Aa^	65.17 ± 0.98^Ab^	71.08 ± 1.04^Aa^	67.94 ± 1.02^Ab^	73.68 ± 0.96^Aa^
2019-2020	W_0_	52.43 ± 1.01^Ab^	55.15 ± 1.03^Aa^	54.80 ± 1.02^Ab^	57.63 ± 1.04^Aa^	64.35 ± 0.85^Ab^	69.66 ± 1.02^Aa^	67.15 ± 0.98^Ab^	72.37 ± 1.05^Aa^
	W_1_	53.38 ± 1.03^Ab^	56.62 ± 0.99^Aa^	55.76 ± 0.95^Ab^	59.08 ± 1.02^Aa^	65.23 ± 0.98^Ab^	71.01 ± 0.99^Aa^	68.07 ± 1.03^Ab^	73.79 ± 0.99^Aa^
	W_2_	54.82 ± 1.02^Ab^	58.21 ± 1.02^Aa^	57.20 ± 1.03^Ab^	60.64 ± 1.03^Aa^	66.59 ± 1.02^Ab^	72.57 ± 1.03^Aa^	69.49 ± 1.02^Ab^	75.30 ± 1.02^Aa^
		Soluble sugar content (%)				Sucrose content (%)			
2018-2019	W_0_	65.07 ± 1.02^Ab^	68.12 ± 1.04^Aa^	63.86 ± 1.01^Ab^	66.81 ± 1.02^Aa^	16.68 ± 0.23^Bb^	18.83 ± 0.20^Ca^	15.96 ± 0.21^Bb^	18.02 ± 0.20^Ca^
	W_1_	66.14 ± 0.97^Ab^	69.33 ± 0.99^Aa^	64.88 ± 0.95^Ab^	67.99 ± 0.97^Aa^	17.15 ± 0.28^Bb^	19.62 ± 0.25^Ba^	16.42 ± 0.20^Bb^	18.84 ± 0.21^Ba^
	W_2_	67.28 ± 1.03^Ab^	70.65 ± 1.01^Aa^	65.98 ± 1.02^Ab^	69.26 ± 1.01^Aa^	18.82 ± 0.26^Ab^	20.37 ± 0.24^Aa^	16.98 ± 0.23^Ab^	19.59 ± 0.19^Aa^
2019-2020	W_0_	66.24 ± 0.94^Ab^	69.45 ± 1.03^Aa^	65.06 ± 0.94^Ab^	68.09 ± 1.03^Aa^	17.03 ± 0.21^Bb^	19.34 ± 0.20^Ca^	16.39 ± 0.25^Bb^	18.64 ± 0.18^Ca^
	W_1_	67.40 ± 1.04^Ab^	70.72 ± 1.00^Aa^	66.20 ± 1.04^Ab^	69.31 ± 0.99^Aa^	17.63 ± 0.22^Bb^	20.41 ± 0.25^Ba^	17.11 ± 0.21^Bb^	19.72 ± 0.22^Ba^
	W_2_	68.59 ± 1.01^Ab^	73.13 ± 0.96^Aa^	67.43 ± 1.02^Ab^	70.65 ± 1.02^Aa^	18.39 ± 0.19^Ab^	21.24 ± 0.29^Aa^	17.81 ± 0.23^Ab^	20.52 ± 0.20^Aa^

Values with different capital letter in the same column are significantly different (α = 0.01). Values with different small letters in the same row are significantly different (α = 0.05). W_0_, no irrigation throughout the entire growing period; W_1_, irrigated at the wintering stage (Feekes 3.0); W_2_, irrigated at both the wintering and greening stage (Feekes 4.0); Se_0_, no Se fertilizer; Se_2160_, 2160 g·ha^-1^ pure Se; BGW-1, black-grained wheat Xihei 88; BGW-2, black-grained wheat Heidali.

Significantly higher grain protein, amylose, amylopectin, total starch, soluble sugar, and sucrose contents were observed for BGW grown in Se_2160_-treated soils under the three water regimes than for BGW grown in the control soils in both seasons (α = 0.05).

### 3.3 Effects of irrigation and Se application on Fe, Zn, Mn, and Cu concentrations in grains

Fe, Zn, and Mn concentrations in grains of BGW grown in control soils were slightly increased by irrigation in both seasons but were significantly increased with Se_2160_ addition (α = 0.01) (**Table 3**). Regardless of the Se treatment, the Cu concentration in grain was not significantly affected by irrigation. When Se_2160_ was added to the soil, the Fe concentration in grain was significantly increased with increasing irrigation amount (α = 0.01), but no significant differences were observed for the Zn or Mn concentration under W_1_ or W_2_ treatment.

**Table 3:** Effect of irrigation on Fe, Zn, Mn, and Cu concentrations of black-grained wheat in different Se soil in two seasons.

Seasons	Treatments	Fe (mg·kg^-1^)				Zn (mg·kg^-1^)			
		BGW-1		BGW-2		BGW-1		BGW-2	
		Se_0_	Se_2160_	Se_0_	Se_2160_	Se_0_	Se_2160_	Se_0_	Se_2160_
2018-2019	W_0_	31.13 ± 1.15^Ab^	37.80 ± 1.06^Ca^	35.25 ± 1.21^Ab^	37.40 ± 0.86^Ca^	33.96 ± 1.25^Ab^	37.25 ± 1.06^Ba^	31.15 ± 1.06^Ab^	34.23 ± 0.80^Ba^
	W_1_	32.21 ± 1.20^Ab^	41.73 ± 0.96^Ba^	36.51 ± 1.17^Ab^	41.06 ± 0.80^Ba^	34.60 ± 1.14^Ab^	40.71 ± 1.14^ABa^	32.31 ± 1.31^Ab^	35.75 ± 1.13^ABa^
	W_2_	33.11 ± 0.96^Ab^	47.21 ± 1.15^Aa^	37.09 ± 1.00^Ab^	44.54 ± 1.07^Aa^	35.90 ± 1.17^Ab^	43.39 ± 1.39^Aa^	33.19 ± 1.28^Ab^	38.11 ± 1.06^Aa^
2019-2020	W_0_	32.68 ± 1.23^Ab^	38.50 ± 0.72^Ca^	36.00 ± 1.25^Ab^	38.44 ± 0.92^Ca^	34.76 ± 1.20^Ab^	37.54 ± 1.05^Ba^	32.09 ± 1.25^Ab^	35.66 ± 0.96^Ba^
	W_1_	33.51 ± 1.29^Ab^	41.87 ± 0.81^Ba^	37.22 ± 1.20^Ab^	42.37 ± 0.85^Ba^	35.30 ± 1.24^Ab^	40.14 ± 0.99^ABa^	33.42 ± 1.07^Ab^	37.97 ± 1.02^ABa^
	W_2_	34.65 ± 1.14^Ab^	48.44 ± 0.91^Aa^	38.03 ± 1.07^Ab^	45.65 ± 0.72^Aa^	36.68 ± 0.89^Ab^	43.02 ± 1.33^Aa^	34.04 ± 1.38^Ab^	41.09 ± 1.25^Aa^
		Mn (mg·kg^-1^)				Cu (mg·kg^-1^)			
2018-2019	W_0_	27.80 ± 1.29^Ab^	31.07 ± 0.97^Ba^	27.09 ± 1.31^Ab^	30.19 ± 0.74^Ba^	8.53 ± 0.68^Aa^	8.33 ± 0.57^Aa^	8.49 ± 0.77^Aa^	8.20 ± 0.56^Aa^
	W_1_	28.44 ± 1.27^Ab^	33.61 ± 1.07^ABa^	28.34 ± 1.20^Ab^	31.76 ± 0.76^ABa^	8.12 ± 0.84^Aa^	7.70 ± 0.61^Aa^	8.14 ± 0.75^Aa^	7.64 ± 0.54^Aa^
	W_2_	29.76 ± 0.77^Ab^	36.44 ± 0.87^Aa^	29.17 ± 0.80^Ab^	33.35 ± 0.83^Aa^	7.38 ± 0.59^Aa^	6.93 ± 0.51^Aa^	7.43 ± 0.62^Aa^	6.92 ± 0.43^Aa^
2019-2020	W_0_	28.80 ± 1.26^Ab^	31.91 ± 0.95^Ba^	28.62 ± 1.07^Ab^	31.67 ± 0.81^Ba^	7.79 ± 0.59^Aa^	7.19 ± 0.89^Aa^	7.69 ± 0.65^Aa^	7.14 ± 0.83^Aa^
	W_1_	29.94 ± 1.17^Ab^	34.66 ± 1.02^ABa^	29.75 ± 1.16^Ab^	33.79 ± 1.10^ABa^	7.45 ± 0.65^Aa^	6.63 ± 0.63^Aa^	7.43 ± 0.73^Aa^	6.73 ± 0.67^Aa^
	W_2_	30.86 ± 1.27^Ab^	37.62 ± 0.61^Aa^	30.53 ± 0.88^Ab^	35.32 ± 1.00^Aa^	6.72 ± 0.61^Aa^	5.91 ± 0.50^Aa^	6.77 ± 0.62^Aa^	5.95 ± 0.48^Aa^

Values with different capital letter in the same column are significantly different (α = 0.01). Values with different small letters in the same row are significantly different (α = 0.05). W_0_, no irrigation throughout the entire growing period; W_1_, irrigated at the wintering stage (Feekes 3.0); W_2_, irrigated at both the wintering and greening stage (Feekes 4.0); Se_0_, no Se fertilizer; Se_2160_, 2160 g·ha^-1^ pure Se; BGW-1, black-grained wheat Xihei 88; BGW-2, black-grained wheat Heidali.

Se_2160_ application significantly increased the Fe, Zn, and Mn concentrations in grains under the three water regimes (W_0_, W_1_, and W_2_) in two seasons compared to Se_0_ application in both seasons (α = 0.05) but did not significantly affect the Cu concentration in grain.

### 3.4 Effects of irrigation and Se application on Se uptake

In control soils, Se uptake of Se in grains, leaves, stem + leaf sheath, and roots of BGW was not significantly affected by irrigation in either season (**Table 4**) but was significantly increased when Se_2160_ was applied to the soil (α = 0.01). Regardless of the Se treatment, irrigation had no significant effect on the Se concentration in the spike-stalk + glume. After applying Se_2160_, the Se concentration in the grains, leaves, and roots of BGW were significantly increased with an increasing irrigation amount (α = 0.01), but no significant differences were found for the stem + leaf sheath between W_1_ and W_2_ treatments. Regardless of the water regime, BGW grown in soils with the addition of Se_2160_ had a significantly higher Se concentration in the 5 plant parts than BGW grown in control soils (α = 0.05). The TF_root-grain_ of BGW grown in Se_2160_-treated soil was increased by irrigation in both seasons, but no significant differences were found among the three water regimes (**Table 4**). Se_2160_ application did not significantly affect TF_root-grain_ under any of the water regimes in either season.

**Table 4:** Effects of irrigation on Se concentrations in different plant parts and the translocation factor from the root to the grain of black-grained wheat in different Se soil in two seasons.

Seasons	Treatments	Grain (mg·kg^-1^)				Spike-stalk + glume (mg·kg^-1^)			
		BGW-1		BGW-2		BGW-1		BGW-2	
		Se_0_	Se_2160_	Se_0_	Se_2160_	Se_0_	Se_2160_	Se_0_	Se_2160_
2018-2019	W_0_	0.061 ± 0.01^Ab^	0.305 ± 0.01^Ca^	0.059 ± 0.01^Ab^	0.292 ± 0.01^Ca^	0.111 ± 0.01^Ab^	0.461 ± 0.02^Aa^	0.102 ± 0.01^Ab^	0.436 ± 0.02^Aa^
	W_1_	0.065 ± 0.02^Ab^	0.370 ± 0.01^Ba^	0.060 ± 0.01^Ab^	0.359 ± 0.02^Ba^	0.113 ± 0.01^Ab^	0.488 ± 0.02^Aa^	0.105 ± 0.01^Ab^	0.459 ± 0.02^Aa^
	W_2_	0.064 ± 0.01^Ab^	0.448 ± 0.03^Aa^	0.061 ± 0.01^Ab^	0.423 ± 0.01^Aa^	0.114 ± 0.01^Ab^	0.538 ± 0.04^Aa^	0.101 ± 0.01^Ab^	0.508 ± 0.03^Aa^
2019-2020	W_0_	0.067 ± 0.01^Ab^	0.294 ± 0.01^Ca^	0.057 ± 0.01^Ab^	0.277 ± 0.01^Ca^	0.102 ± 0.01^Ab^	0.483 ± 0.02^Aa^	0.101 ± 0.01^Ab^	0.445 ± 0.03^Aa^
	W_1_	0.069 ± 0.01^Ab^	0.360 ± 0.01^Ba^	0.059 ± 0.01^Ab^	0.338 ± 0.01^Ba^	0.103 ± 0.01^Ab^	0.504 ± 0.02^Aa^	0.104 ± 0.01^Ab^	0.469 ± 0.02^Aa^
	W_2_	0.070 ± 0.01^Ab^	0.430 ± 0.01^Aa^	0.060 ± 0.01^Ab^	0.395 ± 0.02^Aa^	0.101 ± 0.01^Ab^	0.546 ± 0.01^Aa^	0.103 ± 0.01^Ab^	0.521 ± 0.01^Aa^
		Leaves (mg·kg^-1^)				Stem + leaf sheath (mg·kg^-1^)			
2018-2019	W_0_	0.140 ± 0.01^Ab^	0.558 ± 0.02^Ca^	0.123 ± 0.01^Ab^	0.508 ± 0.02^Ca^	0.116 ± 0.01^Ab^	0.425 ± 0.02^Ba^	0.108 ± 0.01^Ab^	0.316 ± 0.01^Ba^
	W_1_	0.143 ± 0.01^Ab^	0.665 ± 0.01^Ba^	0.125 ± 0.01^Ab^	0.616 ± 0.02^Ba^	0.118 ± 0.01^Ab^	0.501 ± 0.02^Aa^	0.109 ± 0.01^Ab^	0.378 ± 0.02^Aa^
	W_2_	0.144 ± 0.01^Ab^	0.741 ± 0.02^Aa^	0.127 ± 0.01^Ab^	0.688 ± 0.01^Aa^	0.117 ± 0.01^Ab^	0.546 ± 0.02^Aa^	0.107 ± 0.01^Ab^	0.432 ± 0.01^Aa^
2019-2020	W_0_	0.147 ± 0.01^Ab^	0.556 ± 0.01^Ca^	0.120 ± 0.01^Ab^	0.519 ± 0.02^Ca^	0.108 ± 0.01^Ab^	0.391 ± 0.02^Ba^	0.096 ± 0.01^Ab^	0.308 ± 0.01^Ba^
	W_1_	0.150 ± 0.01^Ab^	0.675 ± 0.01^Ba^	0.125 ± 0.01^Ab^	0.605 ± 0.02^Ba^	0.110 ± 0.01^Ab^	0.469 ± 0.02^Aa^	0.097 ± 0.01^Ab^	0.370 ± 0.02^Aa^
	W_2_	0.149 ± 0.01^Ab^	0.765 ± 0.01^Aa^	0.123 ± 0.01^Ab^	0.722 ± 0.01^Aa^	0.107 ± 0.01^Ab^	0.521 ± 0.01^Aa^	0.095 ± 0.01^Ab^	0.416 ± 0.01^Aa^
		Root (mg·kg^-1^)				TF_root-grain_			
2018-2019	W_0_	0.231 ± 0.01^Ab^	1.157 ± 0.01^Ca^	0.231 ± 0.01^Ab^	1.235 ± 0.01^Ca^	0.265 ± 0.02^Aa^	0.264 ± 0.02^Aa^	0.237 ± 0.04^Aa^	0.236 ± 0.02^Aa^
	W_1_	0.223 ± 0.01^Ab^	1.281 ± 0.02^Ba^	0.233 ± 0.01^Ab^	1.373 ± 0.02^Ba^	0.293 ± 0.02^Aa^	0.297 ± 0.01^Aa^	0.258 ± 0.03^Aa^	0.261 ± 0.01^Aa^
	W_2_	0.220 ± 0.02^Ab^	1.450 ± 0.03^Aa^	0.230 ± 0.01^Ab^	1.560 ± 0.01^Aa^	0.291 ± 0.01^Aa^	0.309 ± 0.01^Aa^	0.264 ± 0.02^Aa^	0.271 ± 0.01^Aa^
2019-2020	W_0_	0.242 ± 0.01^Ab^	1.118 ± 0.02^Ca^	0.247 ± 0.01^Ab^	1.281 ± 0.03^Ca^	0.268 ± 0.02^Aa^	0.263 ± 0.02^Aa^	0.214 ± 0.03^Aa^	0.216 ± 0.02^Aa^
	W_1_	0.233 ± 0.01^Ab^	1.231 ± 0.01^Ba^	0.252 ± 0.01^Ab^	1.417 ± 0.01^Ba^	0.298 ± 0.03^Aa^	0.293 ± 0.01^Aa^	0.235 ± 0.04^Aa^	0.239 ± 0.01^Aa^
	W_2_	0.230 ± 0.02^Ab^	1.400 ± 0.04^Aa^	0.250 ± 0.01^Ab^	1.607 ± 0.05^Aa^	0.306 ± 0.04^Aa^	0.307 ± 0.02^Aa^	0.240 ± 0.02^Aa^	0.246 ± 0.01^Aa^

Values with different capital letter in the same column are significantly different (α = 0.01). Values with different small letters in the same row are significantly different (α = 0.05). W_0_, no irrigation throughout the entire growing period; W_1_, irrigated at the wintering stage (Feekes 3.0); W_2_, irrigated at both the wintering and greening stage (Feekes 4.0); Se_0_, no Se fertilizer; Se_2160_, 2160 g·ha^-1^ pure Se; BGW-1, black-grained wheat Xihei 88; BGW-2, black-grained wheat Heidal.

### 3.5 Effects of irrigation and Se application on the Se distribution

**Figure 3:**
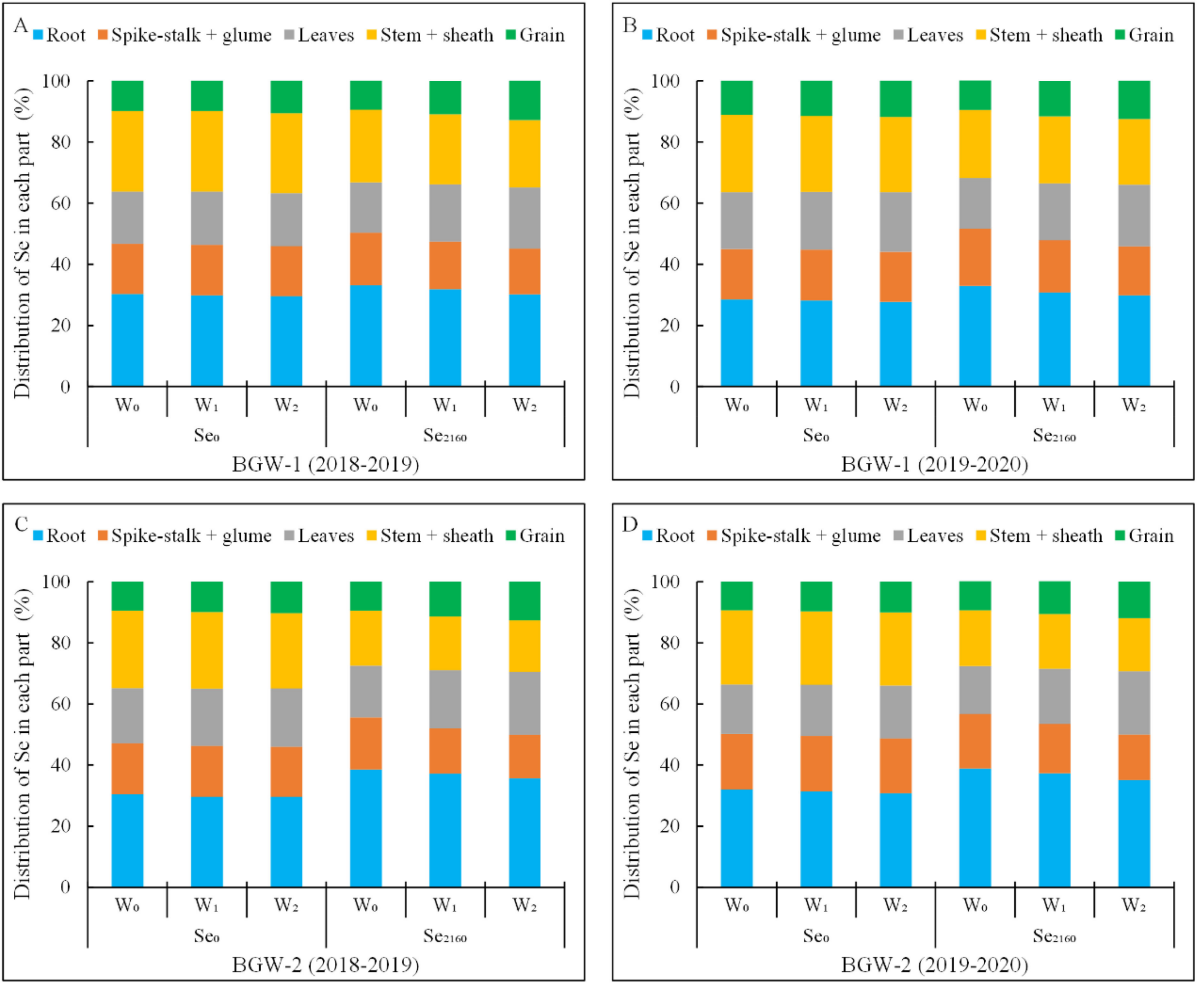
Percentages of Se in spike-stalk + glum, leaves, stem + sheath, and grains compared to that of total Se in black-grained wheat in the two growing seasons.

As shown in **Figure 3**, a large proportion of the total Se (27.8–38.9%) was distributed in roots under all the treatments in both seasons, whereas a small amount of Se (9.3–12.8%) was observed in grains. Irrigation had no significant effect on the Se distribution in each part of BGW grown in control soils, but it significantly decreased and increased the Se distribution in spike-stalk + glume and leaves, respectively, when applying Se_2160_ to the soil (α = 0.01). Moreover, following Se_2160_ application, the Se distribution decreased in roots (BGW-1, W_1_: 31.8% vs. 30.2%, W_2_: 30.8% vs. 29.8%; BGW-2, W_1_: 37.3% vs. 35.7%, W_2_: 37.3% vs. 35.1%) and increased in grains (BGW-1, W_1_: 10.8% vs. 12.8%, W_2_: 11.6% vs. 12.5%; BGW-2, W_1_: 11.4% vs. 12.7%, W_2_: 10.8% vs. 12.0%) with increasing irrigation amounts in both seasons. After Se_2160_ application, there were no significant differences in Se distribution in the stem + sheath among the three water regimes in either season, but the lowest and highest Se distributions in roots and grains, respectively, were observed under W_2_ treatments (α = 0.01).

A significantly higher Se distribution in the roots of BGW-1 (2019–2020) and BGW-2 (both seasons) and a significantly lower Se distribution in the stem + sheath of BGW were observed in Se_2160_-treated soils under the three water regimes than in control soils (*P* = 0.05). Moreover, BGW grown in Se_2160_-treated soils showed a significantly higher Se distribution in grains than BGW grown in control soils under the W_2_ treatment (α = 0.05).

Selenium distribution in each part represents the proportion in the total Se taken up by the whole plant. W_0_, no irrigation throughout the entire growing period; W_1_, irrigated at the wintering stage (Feekes 3.0); W_2_, irrigated at both the wintering and greening stage (Feekes 4.0); Se_0_, no Se fertilizer; Se_2160_, 2160 g·ha^-1^ pure Se; BGW-1, black-grained wheat genotype Xihei 88; BGW-2, black-grained wheat genotype Heidali.

### 3.6 Effects of irrigation and Se application on soil bioavailable Se concentration

In control soil, the bioavailable Se concentration in the 0–20-cm layer was not significantly affected by irrigation but was significantly reduced in Se_2160_-treated soils in both seasons (α = 0.01) (**Table 5**). Moreover, following Se_2160_ application, the soil available Se concentration significantly decreased with an increasing irrigation amount (α = 0.01). The bioavailable Se concentration in the 0–20-cm layer of Se_2160_-treated soil was significantly higher than that in control soil under the 3 water regimes (α = 0.05). Irrigation and Se_2160_ application did not affect the bioavailable Se concentration in the 20–40-cm layer of soil in either season.

**Table 5:** Effects of irrigation on the bioavailable Se concentration in different soil layers at harvest in two seasons.

Seasons	Treatments	Bioavailable Se concentration (μg·kg^-1^) (0–20-cm)			
		BGW-1		BGW-2	
		Se_0_	Se_2160_	Se_0_	Se_2160_
2018-2019	W_0_	16.87 ± 1.31^Ab^	63.88 ± 1.12^Aa^	16.79 ± 1.18^Ab^	65.62 ± 0.95^Aa^
	W_1_	16.63 ± 1.14^Ab^	56.17 ± 1.07^Ba^	16.48 ± 1.11^Ab^	58.17 ± 1.06^Ba^
	W_2_	16.40 ± 1.37^Ab^	44.34 ± 1.63^Ca^	16.31 ± 1.49^Ab^	47.26 ± 1.69^Ca^
2019-2020	W_0_	17.03 ± 1.31^Ab^	64.48 ± 1.29^Aa^	16.95 ± 1.50^Ab^	66.01 ± 1.40^Aa^
	W_1_	16.69 ± 1.16^Ab^	57.33 ± 1.75^Ba^	16.82 ± 1.26^Ab^	58.91 ± 1.08^Ba^
	W_2_	16.78 ± 1.41^Ab^	46.17 ± 1.19^Ca^	16.66 ± 1.65^Ab^	48.33 ± 1.35^Ca^
		Bioavailable Se concentration (μg·kg^-1^) (20–40-cm)			
2018-2019	W_0_	10.89 ± 1.22^Aa^	11.84 ± 1.36^Aa^	10.38 ± 1.31^Aa^	11.40 ± 1.44^Aa^
	W_1_	10.67 ± 1.43^Aa^	11.73 ± 1.57^Aa^	10.08 ± 1.56^Aa^	10.97 ± 1.63^Aa^
	W_2_	10.38 ± 1.11^Aa^	11.61 ± 1.26^Aa^	9.79 ± 1.10^Aa^	10.84 ± 1.25^Aa^
2019-2020	W_0_	10.78 ± 1.29^Aa^	12.06 ± 1.46^Aa^	10.46 ± 1.51^Aa^	11.72 ± 1.71^Aa^
	W_1_	10.49 ± 1.69^Aa^	11.83 ± 1.71^Aa^	10.25 ± 1.13^Aa^	11.34 ± 1.29^Aa^
	W_2_	10.41 ± 1.08^Aa^	11.71 ± 1.27^Aa^	10.08 ± 1.27^Aa^	11.23 ± 1.41^Aa^

Values with different capital letter in the same column are significantly different (α = 0.01). Values with different small letters in the same row are significantly different (α = 0.05). W_0_, no irrigation throughout the entire growing period; W_1_, irrigated at the wintering stage (Feekes 3.0); W_2_, irrigated at both the wintering and greening stage (Feekes 4.0); Se_0_, no Se fertilizer; Se_2160_, 2160 g·ha^-1^ pure Se; BGW-1, black-grained wheat Xihei 88; BGW-2, black-grained wheat Heidali.

## 4 Discussion

### 4.1 Se fertigation enhances the grain yield and kernel number of BGW

Wheat is the second most produced cereal grain worldwide, and it plays an essential role in the human diet. It is also a staple food for nearly half of the Chinese population, where up to 85% of wheat is consumed as flour-derived products. The overall demand for foodstuffs with high nutritional value is increasing due to the increasing prevalence of lifestyle diseases worldwide. In addition to traditional cereal crops, color-grained wheat provides an opportunity for cultivation and processing (Padhy et al., 2022). Feng et al. (2009) reported that irrigation significantly affected grain yield and the 1000- kernel weight of green-grained wheat, and their highest values were found under irrigation treatment (irrigation at fifth leaf emergence, anthesis, and grain-filling stages). The 1000-kernel weight of color-grained wheat significantly differed between irrigation two and three times at 15 and 25 days after anthesis (Yang et al., 2010). Higher antioxidant contents and lower grain yields have been observed for selected pigmented wheat in an organic cropping system and in drier years (Zrckova et al., 2018). Drought stress (drought induced by skipping irrigation at the fourth leaf and anthesis stages) significantly reduced gas exchange parameters and grain yields in wheat (Ejaz et al., 2022). In the present study, the least rainfall was recorded between December and January and between February and March in both years (**Figure 1**), occurring during the wintering and green-turning stages, consistent with the time of irrigation. In this study, the net photosynthetic rate of BGW was remarkably improved by water application. The highest grain yield, spike number, and kernel number were found in BGW at the wintering and green-turning stages. Non-significant differences in the 1000-kernel weight may be due to the amount and timing of irrigation.

Previous studies have reported that Se fertilizer promotes photosynthetic efficiency regarding pigments contents and gas exchange parameters, thereby markedly boosting plant growth and biomass accumulation (Alves et al., 2020). The different results of the net photosynthetic rate observed in this study may have been due to Se fertilizer and its application method. Alves et al. (2020) applied 500 mL of 10 μM sodium selenite solution daily to rice plants during the entire experiment, whereas, in the present study, 2160 g·ha^−1^ Se ore powder was applied and mixed thoroughly with the soil before plowing.

Supplemental Se resulted in a much higher grain yield under normal water stress conditions (Nawaz et al., 2015). Almost all yield and yield components of wheat were significantly increased by exogenous Se application (Se fertigation and Se foliar spraying) (Nawaz et al., 2015). Se application can improve plants’ defense systems by enabling them to recruit and boost beneficial microorganisms in rhizosphere soil, providing further protection (Li et al., 2022). The application of 600 kg·ha^−1^ organic Se fertilizer to soil substantially enhances wheat yield (Chen et al., 2023). In this study, the highest grain yield and its components in BGW were observed with Se_2160_-treated soil under irrigation treatments compared to rain-fed conditions, consistent with previous studies. This may be explained by the improved photosynthetic traits and increased sugar content (as observed in the present study), which contribute to crop growth (Guo et al., 2022). In addition, soil texture, soil physicochemical characteristics, and the method and timing of Se application influence its relative effectiveness in improving crop yield. Irrigation with soil application of Se ore powder maximizes the effect of soil chemistry and microbiology on Se uptake and accumulation, thus improving grain yield and its components.

### 4.2 Se fertigation fortifies the sucrose content and Fe, Zn, and Mn concentrations in BGW grains

Malnutrition, unhealthy diets, and lifestyle changes are the major risk factors for chronic diseases in humans, adversely affecting sustainable development goals. Color-grained wheat and its derived products are key to global nutritional security (Padhy et al., 2022). Biofortification is a strategy employed to produce crop products rich in deficient elements, but the concentrations of other nutrients important for human consumption should not be adversely affected (Sunic and Spanic, 2024). Improved nutrient uptake largely depends on the crop species and its growth environment (Drahoňovský et al., 2016). The total protein content of purple- and green-grained wheat was significantly affected by irrigation (Yang et al., 2010). The differing grain protein contents in the present study may be due to the timing and amount of irrigation. Se supplementation increases mineral uptake and water-soluble protein and sugar contents (Alves et al., 2020). The combination of Se and high nitrogen increased protein concentrations but decreased Fe, Mn, Cu, and Zn concentrations in rice grains (Teixeira et al., 2021). The findings of this study concerning the grain protein content are consistent with those of previous studies, a result that may be due to the increased nitrogen uptake under Se fertilization (Guo et al., 2022). Se fertilizer had a positive effect on carbohydrate accumulation in rice grains (Teixeira et al., 2021), and the mechanism involved carbon fixation, transport, and metabolism. Water-stressed wheat plants fertigated with Se had a higher total soluble sugar content than those under a normal water supply (Nawaz et al., 2015). The soluble sugar content in tomato increased considerably after the application of Se-enriched fertilizer (Huang et al., 2021). Significantly higher soluble sugar and sucrose contents were observed in BGW after Se_2160_ application, possibly due to increased acid invertase activity, because it plays an important role in sugar accumulation (Zhu et al., 2017). Sugar transporter genes may be upregulated in response to Se application (Ren et al., 2022).

Water stress markedly decreased the grain Fe, Zn, and Mg concentrations after exogenous Se supply (Nawaz et al., 2015). Na_2_SeO_4_ as a Se fertilizer application enhanced the quality of water-stressed plants (Nawaz et al., 2015). Water stress reduced the micromineral content in wheat grains (Silva et al., 2020). However, Se-enriched irrigation had little effect on the concentrations of macro- or microelements (Cu, Fe, I, K, Mg, Mn, P, and Zn) in the dry weights of green pea and carrot (Ragalyi et al., 2022). The increased Fe concentration in grains by Se fertigation might be attributed to increased osmoprotectant production or to the activities of catalase, peroxidase, and ascorbate peroxidase (Nawaz et al., 2015). Se facilitates the biosynthesis of pigments, such as chlorophyll, by improving nutrient accumulation, thus benefitting the photosynthetic system (Alves et al., 2020). This could explain the increased Fe, Zn, and Mn concentrations observed in the present study. The Fe, Zn, and Mn concentrations significantly increased in BGW grains due to irrigation after applying Se_2160_ application to the soil, but Cu concentrations in grains showed no positive effects, suggesting that Se ore powder fertigation has synergistic effects on Fe. The concentration of Fe, Zn, and Mn concentrations were increased in BGW grains following irrigation and Se application. This finding was not in accordance with the observations reported by Chen et al. (2023), who observed an increase in the Ca concentration following Se fertilization. This may be attributable to variations in the types of Se fertilizer and growing environments.

### 4.3 Se fertigation enriches Se in various parts of BGW

Se is crucial for human and animal nutrition because of its function as a co-factor in many enzymes (Gupta et al., 2021). In China, approximately 51% of soils are deficient in Se (Dinh et al., 2018), resulting in people in this region experiencing Se deficiency. Therefore, biofortification technology for increasing the Se concentration in crop plants has become a popular research field in recent years.

Se application to staple crops can enhance the Se concentration in grains, thereby meeting the demand for Se in the human diet (Gupta et al., 2021). A proper dose of Se fertilizer increases Se accumulation in different plant parts (Teixeira et al., 2021). Se application significantly increases the Se concentration in wheat organs, and the leaves have the strongest Se accumulation ability, followed by grains, stems, and glumes (Ma et al., 2022). The Se contribution in different parts of the wheat plant after the application of different organic Se fertilizer levels to the soil has been shown to have the following order: roots > grains> ear stem > glume > leaves > stem (Chen et al., 2023). The findings of this study confirmed that soil application of Se ore powder resulted in a much higher Se accumulation in each part of BGW plants. These results are consistent with the findings of Deng et al. (2018), who observed a greater proportion of Se in rice grains and beans after Se ore powder application. The higher Se accumulation in each plant part by Se fertigation may be attributed to the greater abundance of oxidizing bacteria and the improved soil redox environment created by irrigation conditions, which retain more available Se for plants, reducing residual soil Se and increasing the Se concentration in various plant parts (Zhou et al., 2022).

Se primarily accumulates in the roots under selenite fertigation treatments, while Se is largely transported to shoots under selenate fertigation treatments (Zhang et al., 2018). In this study, following soil application of Se_2160_, the irrigation treatments significantly increased the Se concentration in the roots, leaves, and grains of BGW, suggesting that the combination of Se ore powder application and irrigation enhances the Se uptake capacity. Se likely facilitates the response to the irrigation water supply in the expression of sulfate transporter OsSULTR1.2, phosphate transporter OsPT2, and the silicon transporter OsNIP2.1 in roots, thereby improving the Se uptake capacity in roots and increasing the Se concentration in leaves and grains (Teixeira et al., 2021). BGW grown in soil with 2160 g·ha^−1^ Se ore powder was a more efficient Se accumulator under the W_2_ treatment due to Se being readily taken up by the roots, yielding a significantly higher Se concentration in the grains than that under the W_1_ treatment. The potential for Se uptake by wheat in Se-enriched soils is greatly enhanced by irrigation, which is especially important in the presence of elevated water amounts, as this increases Se uptake by BGW. The use of irrigation both during the wintering and greening stages in soil with Se_2160_ addition did caused Se enrichment in BGW, thus, it is recommended to be used for biofortification. Se-enriched BGW could be useful as a raw material in Se-deficient areas where the population largely depends on cereal foods. The results of this study confirm that irrigation after Se_2160_ application can not only achieve BGW with high grain yield but also produce higher grain nutritional quality.

### 4.4 Se fertigation improves the bioavailable Se concentration in the 0–20-cm soil layer

The edible parts of crop plants are the main sources of dietary Se, while the Se concentration in crops is determined by Se bioavailability in the soil. Most Se in the soil is not available to plants (Ali et al., 2017). Se^4+^, Se^6+^, and elemental Se (Se^0^) are the main forms distributed in soil (Guo et al., 2022). Among these, Se^4+^ and Se^6+^ are the main Se fractions determining the bioavailable Se concentrations in the soil. Se fractions are separated into soluble Se, exchangeable Se, Fe/Mn oxide-bound Se, organic matter-bound Se, and residual Se (Ali et al., 2017). However, the distribution patterns of Se fractions in soil are in a state of dynamic equilibrium regulated by adsorption/desorption, precipitation/dissolution, and oxidation/reduction processes (Guo et al., 2022). The intensity of these transformation processes is controlled by soil properties, such as soil texture, moisture, pH, redox conditions, organic matter, and microbial functions (Guo et al., 2022).

Flooded environments promote residual Se transformation into Fe/Mn oxide-bound Se, and thus increasing the Se activity of Se in the soil (Deng et al., 2018). Flood irrigation reduced the bioavailability of soil Se, and the concentration of water-soluble and ion-exchangeable Se was reduced from 8.0% to 5.0% of the total Se (Song et al., 2020). Se in the soil of riverside flood plains can be transformed into water-soluble and ion-exchangeable Se (VI) (Zhang et al., 2023). Moreover, water retention regulation and organic matter transport affect Se speciation and partitioning in the soil of mollisol lands far from river channels. Se fertigation with nanobubbles reduces soil Se accumulation and increases the Se content in cucumber (Zhou et al., 2022). The results of this study confirmed our hypothesis that irrigation and increasing irrigation amount would decrease soil residual Se in the 0–20-cm layer following Se_2160_ application, ultimately improving wheat grain yield and quality. This result may be ascribed in part to the transformation of organic-bound Se and infiltrated oxygenated water, which increase water-soluble and ion-exchangeable Se (Zhang et al., 2023). By analyzing the variation in the soil bioavailable Se concentration in the 0–20-cm layer under the same water regimes, we found that Se ore powder application promoted soil bioavailable Se accumulation. Se in ore powder is mainly present as Se^4+^, and Se^0^ is formed at a ratio of 4:6 (Deng et al., 2018). Thus, changes among valence states caused by the soil application of Se ore powder can affect Se fractions because of their differences in migration mobility and binding intensity, further affecting Se bioavailability in the soil.

## 5 Conclusions

Se ore powder application and irrigation frequency are important for biofortification of color-grained wheat due to Se scarcity in soil. BGW grown in soil with Se_2160_ addition had a high grain yield and yield component performance when irrigated at the wintering and green-turning stages and thus could be recommended for cropping in Se-deficient soils. The experiment also indicated that irrigation and increasing the irrigation amount significantly increased the sucrose content, grain Fe concentration, and Se concentration in BGW plants after applying Se_2160_ to the soil. Grain yield, nutritional components, and Fe, Zn, and Mn concentrations in BGW grains were enhanced by Se_2160_ application compared to the control. BGW was generally a more efficient Se accumulator in soil treated with Se_2160_. Furthermore, bioavailable Se concentrations in the 0–20-cm layer of Se_2160_-treated soil were significantly decreased as the irrigation amount increased but were significantly higher than those of control soils, suggesting that irrigation is beneficial for the development of Se-enriched agriculture. The experiment also confirmed that Se ore powder application to soil compared to irrigation at the wintering and green-turning stages could be considered the most effective methodologies for Se enrichment of BGW.

## Data Availability

The original contributions presented in the study are included in the article/supplementary material. Further inquiries can be directed to the corresponding author.
